# Association Between Psychosocial Stress and Premature Ventricular Contractions During the Recovery Phase Following Treadmill Testing in Asymptomatic Individuals

**DOI:** 10.3390/jcm14134637

**Published:** 2025-06-30

**Authors:** João Paulo de Almeida Dourado, Luan Morais Azevêdo, Larissa de Almeida Dourado, Jaciara Gomes de Oliveira, Bianca Barros de Faria, Karolyne de Oliveira Matos, Leonardo Roever, Paulo Magno Martins Dourado, Pedro Gabriel Senger Braga

**Affiliations:** 1 Pro-Coracao Clinic, Sao Paulo 05021-010, Brazil; 2 Research in Exercise and Nutrition for Health and Sports Performance, Campo Grande 79080-730, Brazil; 3 Department of Clinical Research, Brazilian Evidence—Based Health Network, Uberlândia 38400-384, Brazil; 4 Cardio-Oncology Department, InCor, Universidade de São Paulo, São Paulo 05403-900, Brazil

**Keywords:** exercise test, premature ventricular contractions, asymptomatic adults, psychosocial stress, cardiovascular risk assessment

## Abstract

**Introduction**: Ventricular arrhythmias may lead to sudden cardiac death and, when occurring during the recovery phase after exercise testing, are associated with increased cardiovascular risk. Aim: To investigate the association between psychosocial stress and the risk of premature ventricular contractions (PVCs) during the recovery phase after treadmill testing in asymptomatic individuals. **Methods**: A total of 282 asymptomatic adults underwent treadmill testing. Participants were categorized into a stress-present group (+S, n = 176) or a stress-absent group (−S, n = 106) based on their self-reported psychosocial stress levels. Inclusion criteria included exercising for at least 6 min and reaching at least 85% of the age-predicted maximum heart rate. Exclusion criteria comprised pre-exercise VAs, unreadable ECGs, chronic medication use, systolic blood pressure ≥180 mmHg, and diastolic blood pressure ≥110 mmHg. This study was registered on ClinicalTrials.gov (NCT05987891). **Results**: Compared to the −S group, the +S group had a higher body mass index (BMI) (*p* = 0.0025); 26.5 (23.9; 29.0) and larger waist circumference (*p* = 0.0001); 95 (86; 103), and reported lower physical activity levels (*p* = 0.0004). Notably, only psychosocial stress and BMI were statistically associated with PVCs during the recovery phase, immediately following the stress test. For each 1 kg/m^2^ increase in BMI, the risk of PVCs decreased by 9%. Participants reporting psychosocial stress had a 9.03-fold higher risk of PVCs compared to those who did not report stress. **Conclusions**: Self-reported psychosocial stress significantly increases the risk of PVC occurrence during the recovery phase of treadmill exercise testing in asymptomatic individuals. These findings may support the development of improved PVC detection strategies and enhance cardiovascular risk assessment in clinical settings.

## 1. Introduction

The global burden of cardiovascular disease (CVD) continues to rise, driven by population aging, lifestyle changes, and increasing prevalence of associated comorbidities [1]. While biochemical markers and imaging techniques have significantly improved a comprehensive cardiovascular assessment, as they are crucial for detecting early-stage disease [2]. Ventricular arrhythmias (VAs) represent a particularly concerning manifestation of CVD due to their association with sudden cardiac death, which often serves as the first clinical presentation in previously asymptomatic individuals [3,4].

Premature ventricular contractions (PVCs) are among the most common arrhythmias detected during clinical evaluation and may serve as early indicators of underlying cardiovascular pathology [5]. PVCs occurring during the recovery phase of exercise stress testing warrant special attention, as they have been associated with significantly higher risk of cardiovascular mortality compared to PVCs occurring during exercise [6,7]. In population studies, PVCs have been identified in 2.4% to 20% of individuals assessed using surface ECG, with prevalence varying based on age, sex, and comorbidities [8,9]. However, standard resting ECGs may underestimate PVC burden due to their brief recording duration. Exercise testing provides a more comprehensive evaluation window and may reveal arrhythmias that remain undetected at rest [10,11]. Furthermore, the transition from exercise to recovery represents a period of significant autonomic flux, characterized by parasympathetic reactivation and sympathetic withdrawal, which may create a vulnerable period for arrhythmogenesis [12].

Psychosocial stress has emerged as a significant modifiable risk factor for cardiovascular disease [13]. Chronic stress exposure activates neurohormonal pathways, including the sympathetic nervous system and hypothalamic–pituitary–adrenal axis, leading to heightened cardiovascular reactivity, endothelial dysfunction, and increased inflammatory responses [14]. These physiological alterations may create a proarrhythmic substrate, particularly during periods of autonomic fluctuation such as exercise recovery [15]. Prior research has demonstrated that stress increases arrhythmia susceptibility through multiple mechanisms, including altered calcium handling in cardiomyocytes, increased oxidative stress, and autonomic imbalance [15].

Despite the established link between psychological factors and cardiovascular outcomes, limited research has examined the specific relationship between self-reported psychosocial stress and recovery-phase PVCs in otherwise healthy individuals. The primary objective of this study is to propose that self-reported stress may contribute to the clinical investigation of cardiovascular conditions, complementing classical cardiovascular symptoms such as fatigue, chest pain, and shortness of breath, among others. Therefore, the present study aims to evaluate the influence of self-reported psychosocial stress, measured as a dichotomous variable, on the occurrence of PVCs during the recovery phase of treadmill exercise testing in asymptomatic individuals.

## 2. Methods

### 2.1. Study Design and Participants

**Figure 1:**
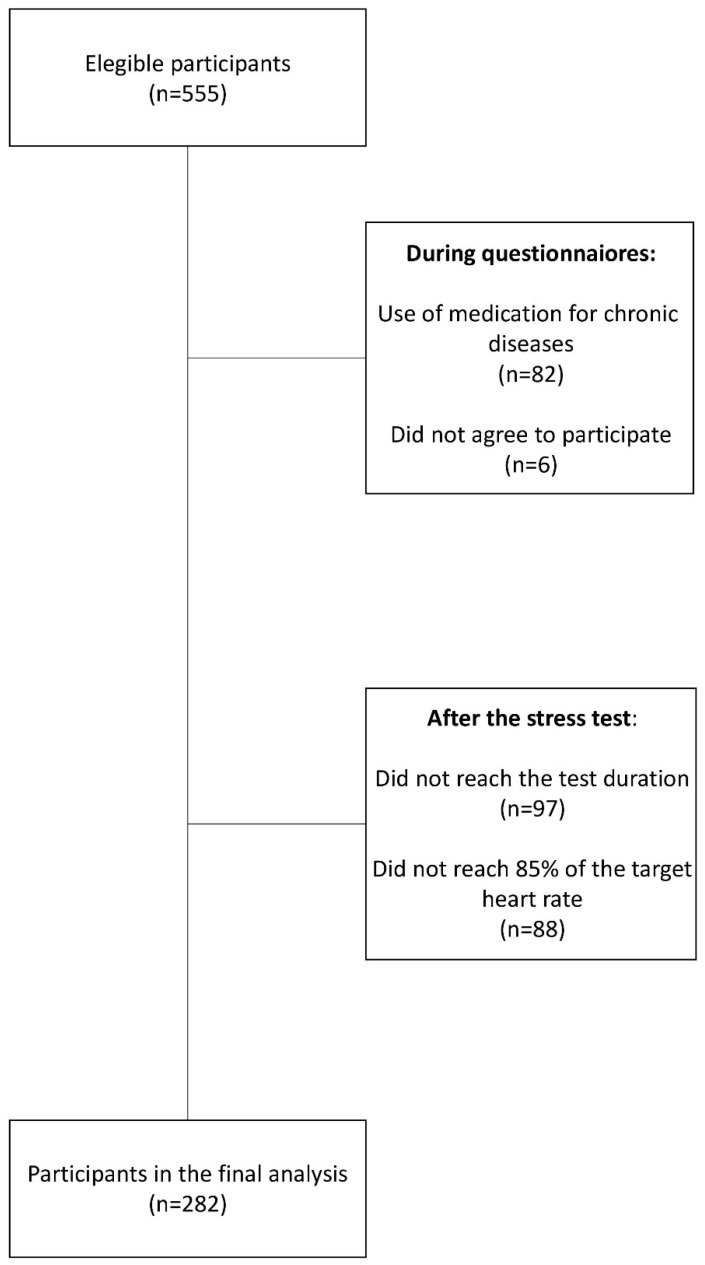
Flowchart of study participants.

A total of 282 asymptomatic participants, of both sexes, aged 18 to 59 years, were enrolled in this cross-sectional study, as outlined in the flowchart Figure 1. Recruitment was conducted at the Pro-Coração clinic, where participants underwent a detailed evaluation of their medical history and a treadmill exercise test. This study was registered at ClinicalTrials.gov (NCT05987891) and approved by the Ethical Committee of the Clinical Hospital of the University of São Paulo Medical School on 24th August 2023 (approval number: 6.258.413). Written informed consent was obtained from all participants prior to enrollment. The investigations were conducted by the principles outlined in the Declaration of Helsinki (1975, revised in 2013). Inclusion and Exclusion Criteria:

The inclusion criteria were: (1) achievement of at least submaximal heart rate (HR) values (≥85% of age-predicted maximum HR), (2) completion of a minimum of six minutes in the effort phase, and (3) performance of the test using the Ellestad protocol.

The exclusion criteria were: (1) presence of VA documented during the rest phase before the exercise test, (2) unreadable ECG, (3) use of medications for chronic diseases, (4) systolic blood pressure (SBP) ≥180 mmHg or diastolic blood pressure (DBP) ≥110 mmHg, (5) ischemic-related changes on ECG, (6) consumption of caffeine or stimulants before the test, and (7) fasting state at the time of testing.

To exclude cardiovascular disease, a stress electrocardiogram was evaluated during a treadmill exercise test in conjunction with a detailed review of the patient’s clinical history.

### 2.2. Psychosocial Stress and Lifestyle Assessment

Psychosocial stress was assessed as a dichotomous variable (present or absent) based on participants’ self-reported responses to a standardized question: “Do you consider yourself to be experiencing significant stress in your daily routine?”, during the cardiologist consultation. This approach aligns with previous research demonstrating that simple self-report measures of perceived stress correlate with more comprehensive stress assessments and predict cardiovascular outcomes [16,17]. Participants who confirmed the presence of stress were allocated to the +S group (n = 176), while those without stress were assigned to the −S group (n = 106).

Participants also reported whether they engaged in 150 min or more of physical activity per week, by World Health Organization recommendations [18]. Smoking habits, including current and former smoking status, were documented using standardized questionnaires.

### 2.3. Familial Cardiovascular Risk Factors

A familial risk factor was considered positive if first-degree relatives had been diagnosed with or died from myocardial infarction, heart attack, or cerebrovascular disease before the age of 60 years, consistent with established cardiovascular risk assessment protocols [5,19].

### 2.4. Treadmill Exercise Testing Protocol

The treadmill test is part of the routine cardiovascular assessment in adults, symptomatic or not, highlighting the importance of primary cardiovascular prevention. Participants underwent an exercise test following either the Bruce or Ellestad protocol based on individual fitness levels and clinical considerations [20,21]. The attending cardiologist made protocol selection based on self-reported physical activity level and age. Participants abstained from caffeine, alcohol, and strenuous exercise for at least 24 h before testing. All tests were conducted in a temperature-controlled environment (21–23 °C) between 8:00 and 11:00 AM to minimize diurnal variations in cardiovascular responses.

Standard 12-lead ECG electrodes were placed according to Mason–Likar placement, with blood pressure measured using a calibrated automated sphygmomanometer (Welch Allyn). Baseline measurements were taken after a 5-min rest in a standing position. During exercise, heart rate, blood pressure, and ECG were continuously monitored, with specific recordings at rest, maximal effort, and during recovery at minutes 1, 2, 4, and 6 [11]. Blood pressure was measured during the last 30 sec of each exercise stage and at specified recovery times, with participants maintaining a slow walking pace (1.0 mph, 0% grade) throughout recovery [22].

Testing continued until participants reached ≥85% of the age-predicted maximum heart rate (220-age) or until predetermined termination criteria were met, including patient request, severe fatigue, signs of poor perfusion, blood pressure abnormalities, technical difficulties, ECG changes, angina, or excessive hypertensive response [23,24]. The standardized 6-min recovery included continuous ECG monitoring and BP measurements at minutes 1, 2, 4, and 6 [23] to observe and document the occurrence of premature ventricular contractions. The delta HR was calculated as the difference between peak HR and HR at the first, second, third, and fourth minutes of the recovery phase, respectively. All exercise tests were conducted and analyzed by the same cardiologist to ensure consistency and minimize inter-observer variability. Nevertheless, the report underwent review by three independent cardiologists (JPAD, LAD, and PMMD).

### 2.5. Premature Ventricular Contractions

PVCs were identified as extra beats characterized by a widened QRS complex, premature timing, and different morphology from the normal QRS complex [8]. The hallmark features of an ECG include a premature and widened QRS complex (greater than 120 ms) without a preceding P wave, indicating a ventricular origin. In addition, PVCs display a compensatory pause—which can help to identify them but is not mandatory—allowing the underlying rhythm to return to its regular cadence. In this study, we included ventricular ectopy in general, which may manifest as premature ventricular contractions that can occur singly, in pairs, triplets, or as runs of ventricular tachycardia [25]. Similarly, various types of ventricular ectopy were not within the scope of this study. The identification of PVCs was analyzed by three cardiologists (JPAD, LAD, and PMMD).

### 2.6. Equity, Diversity, and Inclusion (EDI) Statement

We aimed to ensure equity, diversity, and inclusion in the recruitment and analysis phases of this study. Participants were Brazilian individuals from diverse ethnic backgrounds, reflecting the population’s demographic variability. Care was taken to avoid bias in participant selection and data interpretation, and the analysis considered cultural and contextual factors relevant to stress and PVC risk in Brazil. The research team is committed to inclusive scientific practices and equitable representation in health research. Nevertheless, data regarding ethnicity were not documented in the present study.

### 2.7. Statistical Analysis

The evaluated measures were described for all patients using both absolute and relative frequencies for qualitative characteristics, as well as summary measures (mean, standard deviation, median, and quartiles) for quantitative characteristics. The qualitative characteristics were described in terms of the presence of stress, using both absolute and relative frequencies, and associations were verified using chi-square tests or Fisher’s exact tests. Quantitative characteristics were described according to the presence of stress using summary measures and compared using unpaired Student’s *t*-tests.

The occurrence of arrhythmia during recovery was described according to each qualitative characteristic, and associations were verified using chi-square tests or exact tests (Fisher’s exact test or likelihood ratio test). Quantitative characteristics were described according to the occurrence of arrhythmia during recovery and compared using unpaired Student’s *t*-tests. Unadjusted odds ratios (ORs) with their respective 95% confidence intervals were estimated for each characteristic occurrence of arrhythmia during recovery using simple logistic regression. A multivariate model was adjusted to include the characteristics that showed significance between patients with and without stress and that presented a significance level of less than 0.10 (*p* < 0.10) in the unadjusted analyses related to the occurrence of arrhythmia during recovery to verify whether stress independently influences arrhythmia during recovery regardless of other patient characteristics. It was performed to avoid overfitting and ensure better generalization of the model.

The analyses were performed using IBM SPSS for Windows, version 22.0, and the results were tabulated using Microsoft Excel 2013. The tests were conducted with a significance level of 5%.

## 3. Results

According to Table 1, the +S group exhibited considerably higher BMIs (*p* = 0.025) and waist circumferences (*p* = 0.001). Additionally, the frequency of physical activity was significantly lower compared to the –S group (*p* = 0.004).

As shown in Table 2, heart rate (HR), systolic blood pressure (SBP), and diastolic blood pressure (DBP) were comparable between groups during the resting and exercise phases. However, during the recovery phase, SBP at the sixth minute was significantly higher in the +S group (*p* = 0.0195). Other hemodynamic variables during the recovery phase did not differ significantly between groups. The duration of the exercise test was significantly shorter in the +S group (*p* < 0.0001). Delta HR values at the first, second, fourth, and sixth minutes of recovery were similar between groups.

As presented in Table 3, the occurrence of premature ventricular contractions (PVCs) was significantly associated only with psychosocial stress (*p* < 0.001). Recovery-phase arrhythmias were not influenced by age, sex, body mass index, history of physical activity, abdominal circumference, smoking status, family risk factors, or the presence of ischemia during either exercise or recovery. As shown in Table 4, PVCs were analyzed in a multiple logistic regression model using traditional risk factors, such as age, BMI, physical activity history, and psychosocial stress, the focus of this study. It was observed that BMI was also associated with PVC occurrence (*p* = 0.028). Notably, for each 1 kg/m^2^ increase in BMI, there was a 9% reduction in the odds of experiencing PVCs during the recovery phase. Stressed patients were 9.03 times more likely to present PVCs during recovery compared to those who did not report stress.

## 4. Discussion

This study demonstrated that self-reported psychosocial stress increases the chance of PVCs during the recovery phase of exercise testing in asymptomatic individuals. Our findings contribute to the growing body of evidence supporting the connection between psychological factors and arrhythmogenesis, particularly during periods of autonomic transition such as post-exercise recovery.

The role of psychosocial stress in promoting arrhythmias has been attributed to increased sympathetic nervous system activity and autonomic imbalance [26]. Sympathetic activation can lead to altered calcium handling in cardiomyocytes, increased triggered activity, and enhanced automaticity in ventricular tissue [12]. Furthermore, chronic stress exposure induces structural and electrical remodeling of the myocardium, creating a substrate for the development of arrhythmias [16,27]. Our findings highlight the clinical significance of monitoring PVCs occurring during the recovery phase, as they may identify individuals at increased cardiovascular risk even when asymptomatic.

Interestingly, despite the established influence of the autonomic nervous system on arrhythmogenesis, our assessment using delta HR recovery values showed no significant differences between the study groups. This suggests that mechanisms beyond simple heart rate recovery dynamics might be contributing to stress-induced arrhythmogenesis in our cohort. Alternative pathways could involve altered baroreceptor sensitivity, endothelial dysfunction, or inflammatory processes associated with chronic stress exposure [14]. Furthermore, the observed higher systolic blood pressure at 6 min of recovery in the stressed group may reflect persistent sympathetic activation or impaired vascular relaxation, both of which have been linked to increased arrhythmia susceptibility [28].

The significant difference in exercise duration between the two groups deserves particular attention. Participants who reported psychosocial stress completed shorter exercise tests, despite achieving similar maximum heart rates as the non-stressed group. This finding aligns with previous research demonstrating reduced exercise capacity in individuals experiencing chronic stress [29]. Potential explanations include stress-related fatigue, altered pain perception, reduced motivation, or subclinical cardiovascular dysfunction [30]. The shorter exercise duration in stressed individuals may also reflect differences in physical fitness, in parallel with the lower frequency of physical activity reported by this group.

Our simple screening approach for psychosocial stress demonstrated significant discriminatory power for identifying individuals at risk for recovery-phase PVCs. This finding supports the value of incorporating brief psychological assessments into routine cardiovascular evaluations [31]. PVCs have been established as early indicators of future cardiovascular disease [32], heart failure, and stroke [33]. Premature ventricular contractions were also observed in the different populations, which suggests an interesting point: the occurrence of arrhythmias post-exercise does not necessarily require reaching high heart rate values, as arrhythmias may manifest during more specific tests [34]. Therefore, identifying factors that predispose to PVCs, such as psychosocial stress, may enhance risk stratification in asymptomatic populations.

The anthropometric differences observed between our study groups deserve consideration. Participants in the stress-positive group exhibited significantly higher BMI and waist circumference, consistent with established associations between psychosocial stress and weight gain [27]. Increased adiposity, particularly visceral fat accumulation, promotes systemic inflammation and oxidative stress, which may contribute to arrhythmia susceptibility [35]. However, it is noteworthy that both groups fell within the overweight BMI category (25–29.9 kg/m^2^), suggesting that the differences in PVC frequency cannot be attributed solely to obesity-related mechanisms. On the other hand, our analysis shows that the increase in BMI may be protective against PVC, which may contribute to the obesity paradox [36]. Our finding becomes paradoxical because obesity is well established as a risk factor for cardiac arrhythmias in the literature, including its mechanisms: (electromechanical dysfunction, atrial and ventricular remodeling), as well as epicardial fat, which impairs cardiac electrophysiology by promoting a proarrhythmic substrate [37]. On the other hand, we cannot overlook that the exercise test duration was shorter in stressed individuals, which may reflect their higher BMI and consequently poorer physical condition.

Several limitations of our study warrant acknowledgment. First, our dichotomous assessment of psychosocial stress, while practical for clinical settings, may not capture the complex and multidimensional nature of stress experiences. Future studies could incorporate validated stress questionnaires (e.g., Perceived Stress Scale) or stress-related biomarkers to allow for a more nuanced characterization, alongside a more detailed assessment of arrhythmias. Additionally, our group has a broad interest in analyzing Holter recordings, and we intend to enhance our methodological approach, beginning with the present study. Second, the cross-sectional design precludes the determination of causality between stress and PVCs. Third, we did not assess long-term outcomes, which would be valuable for determining the prognostic significance of stress-associated PVCs. Finally, our sample was recruited from a single center, which may limit its generalizability.

Despite these limitations, our findings have important clinical implications. The increased chance of PVCs in self-reported stress suggests that incorporating simple stress assessments into pre-exercise evaluation may enhance the detection of individuals at increased arrhythmic risk. The questioning about stress may have been superficial; however, it is essential to note that the patients reported complaints about their stressful routines, which is why they sought healthcare services for cardiological evaluation. Furthermore, stress management interventions may represent a potential therapeutic target for reducing PVC burden, although intervention studies are needed to confirm this hypothesis. This sample was composed by convenience, based on individuals who sought healthcare services. Additionally, participants were recruited consecutively. Another limitation is the lack of documented ethnic background for the studied sample.

Concerning clinical implications, this study, along with current evidence, demonstrates the detrimental effect of psychosocial stress on the heart, and our data furnish a number, whereby psychosocial stress increases the risk of PVCs by 9 times. Therefore, understanding which strategies may reduce its impact is crucial for improving outcomes, such as psychotherapy, exercise, and medicines. Additionally, evaluating whether these patients report more symptoms in conjunction with stress can improve risk stratification, such as anxiety.

## 5. Conclusions

In conclusion, self-reported psychosocial stress is associated with an increased likelihood of premature ventricular contractions (PVCs) during the recovery phase following treadmill exercise testing in asymptomatic individuals. This elevated risk was observed despite similar heart rate recovery profiles between stressed and non-stressed participants, suggesting the involvement of mechanisms beyond conventional autonomic markers. Additionally, we found that higher BMI may be protective against the occurrence of PVCs.

Our findings underscore the importance of incorporating psychological factors into cardiovascular risk assessment and suggest that simple screening for psychosocial stress may enhance the identification of subclinical arrhythmic risk in apparently healthy individuals. Future research should investigate whether stress-reduction interventions can influence PVC burden and related cardiovascular outcomes.

## Figures and Tables

**Table 1:** Description of the evaluated characteristics according to the presence of stress and results of statistical tests.

Variable	Psychosocial Stress		*p*
	No (n = 106)	Yes (n = 176)	
Age (years)			0.089 **
Mean ± SD	37.4 ± 10.6	39.7 ± 10.7	
median (p25; p75)	38 (29; 45.3)	40 (32; 48)	
Sex, n (%)			0.640
Female	38 (35.8)	68 (38.6)	
Male	68 (64.2)	108 (61.4)	
BMI (kg/m^2^)			0.025 **
Mean ± SD	26 ± 3.9	27.2 ± 4.3	
median (p25; p75)	25.6 (23.6; 28.2)	26.7 (24.4; 29.2)	
Physical Activity (150 min/sem) n (%)			0.001
No	37 (34.9)	97 (55.1)	
Yes	69 (65.1)	79 (44.9)	
Waist Circumference (cm)			0.004 **
Mean ± SD	91.9 ± 13.2	96.7 ± 13.2	
median (p25; p75)	90 (82; 100.5)	97 (89; 104)	
Smoking			0.288
No	92 (86.8)	140 (79.5)	
Former smoke	7 (6.6)	16 (9.1)	
Current smoke	7 (6.6)	20 (11.4)	
Familial Risk Factor n (%)			0.966
No	69 (65.1)	115 (65.3)	
Yes	37 (34.9)	61 (34.7)	

Abbreviations: BMI: body mass index; Chi-square test; ** Unpaired Student’s *t*-test.

**Table 2:** Hemodynamic variables from study groups.

Parameters/Groups	Psychosocial Stress		*p*
	Yes (n = 176)	No (n = 106)	
Rest			
HR (bpm)	78 ± 13	77 ± 13	0.3214
SBP (mmHg)	120 ± 8	120 ± 7	0.6792
DBP (mmHg)	79 ± 6	78 ± 6	0.2739
MBP (mmHg)	89 ± 19	84 ± 26	0.1049
Maximal			
HR (bpm)	167 ± 12	169 ± 13	0.1846
SBP (mmHg)	151 ± 12	151 ± 11	0.9079
DBP (mmHg)	79 ± 6	78 ± 26	0.3093
MBP (mmHg)	99 ± 21	94 ± 29	0.2232
Recovery			
HR 1 min (bpm)	140 ± 15	141 ± 17	0.7719
HR 2 min (bpm)	121 ± 17	122 ± 17	0.7446
HR 4 min (bpm)	108 ± 14	108 ± 17	0.6965
HR 6 min (bpm)	104 ± 13	104 ± 15	0.7649
SBP 1 min (mmHg)	150 ± 12	150 ± 11	0.7601
SBP 2 min (mmHg)	147 ± 12	146 ± 12	0.9307
SBP 4 min (mmHg)	139 ± 11	138 ± 12	0.9946
SBP 6 min (mmHg)	133 ± 10	129 ± 12	0.0195
DBP 1 min (mmHg)	79 ± 6	78 ± 7	0.1684
DBP 2 min (mmHg)	79 ± 6	78 ± 7	0.1684
DBP 4 min (mmHg)	79 ± 6	78 ± 7	0.0889
DBP 6 min (mmHg)	79 ± 6	77 ± 10	0.0598
Test time (seconds)	435 ± 101	503 ± 154	<0.0001

Abbreviations: BMI: body mass index; WC: waist circumference; HR: heart rate; SBP: systolic blood pressure; DBP: diastolic blood pressure; MBP: mean blood pressure.

**Table 3:** Description of arrhythmia occurrence during recovery according to characteristics of interest and results of unadjusted statistical analyses.

Variable	PVC Recovery Phase		OR	CI (95%)		*p*
	No	Yes		Inferior	Superior	
Age (years)			1.01	0.98	1.04	0.581 **
Mean ± SD	38.7 ± 10.3	39.6 ± 13				
median (p25; p75)	39 (31; 46)	38.5 (28.8; 49.5)				
Sex, n (%)						0.813
Female	88 (83)	18 (17)	1.00			
Male	148 (84.1)	28 (15.9)	0.93	0.48	1.77	
BMI (kg/m^2^)			0.94	0.86	1.02	0.109 **
Mean ± SD	26.9 ± 4.2	25.8 ± 4.3				
median (p25; p75)	26.5 (23.9; 29)	25.5 (22.4; 28.7)				
Physical Activity (150 min/sem.)?, n (%)						0.489
No	110 (82.1)	24 (17.9)	1.00			
Yes	126 (85.1)	22 (14.9)	0.80	0.43	1.51	
Waist Circumference (cm)			0.99	0.97	1.01	0.344 **
Mean ± SD	95.3 ± 12.5	93.2 ± 16.9				
median (p25; p75)	95 (86; 103)	93 (84; 104.3)				
Smoking						0.250 #
No	195 (84.1)	37 (15.9)	1.00			
Former smoke	21 (91.3)	2 (8.7)	0.50	0.11	2.23	
Current smoke	20 (74.1)	7 (25.9)	1.85	0.73	4.67	
Familial Risk Factor, n (%)						0.996
No	154 (83.7)	30 (16.3)	1.00			
Yes	82 (83.7)	16 (16.3)	1.00	0.52	1.94	

Chi-square test; # Likelihood ratio test; ** Unpaired Student’s *t*-test.

**Table 4:** Results of the multiple models explaining the occurrence of arrhythmia during recovery according to the characteristics of interest.

Variable	OR	IC (95%)		*p*
		Inferior	Superior	
Age (years)	1.00	0.97	1.03	0.903
BMI (kg/m^2^)	0.91	0.83	0.99	0.028
Reach Physical Activity Recommendations (150 min/week)	0.98	0.50	1.91	0.941
Psychosocial Stress	9.03	3.07	26.54	<0.001

Multiple Logistic Regression; Abbreviations: BMI: body mass index.

## Data Availability

The datasets used and/or analyzed during the current study are available from the corresponding author upon reasonable request.

## Abbreviations

BMI	body mass index
CVD	cardiovascular disease
ECG	Electrocardiogram
SBP	systolic blood pressure
HR	heart rate
DBP	diastolic blood pressure
MBP	mean blood pressure
PVC	premature ventricular contractions
VA	ventricular arrhythmia
